# Effectiveness of a mesotherapy protocol vs. an epidural injection of platelet-rich plasma or methylprednisolone in degenerative lumbosacral stenosis in dogs – a retrospective study

**DOI:** 10.3389/fvets.2026.1851477

**Published:** 2026-09-16

**Authors:** Tiago Reis, J. C. Alves

**Affiliations:** 1 Faculty of Veterinary Medicine, Lusófona University, Lisbon, Portugal; 2 Divisão de Medicina Veterinária, Guarda Nacional Republicana (GNR). Rua Presidente Arriaga, Lisbon, Portugal; 3 I-MVET, Faculty of Veterinary Medicine, Lisbon University Centre, Lusófona University, Lisbon, Portugal; 4 Centro de Ciência Animal e Veterinária, Lusófona University, Lisbon, Portugal

**Keywords:** degenerative lumbosacral stenosis, dog, epidural, local intradermal therapy, mesotherapy, methylprednisolone, pain, platelet-rich plasma

## Abstract

**Objective:**

This study aimed to compare the effectiveness of an intradermal mesotherapy protocol, an intra-articular injection of platelet-rich plasma (PRP), and an epidural injection of methylprednisolone in managing degenerative lumbosacral stenosis in dogs.

**Study design:**

Retrospective study.

**Animals:**

55 dogs.

**Methods:**

The animals were divided into three treatment groups: a mesotherapy group (MG, *n* = 30) treated with a combination of lidocaine, dexamethasone, and midazolam; a PRP group (PRPG, *n* = 15) treated with epidural PRP; and a methylprednisolone group (MPG, *n* = 10) treated with epidural methylprednisolone acetate. All treatments consisted of two sessions spaced 15 days apart. Clinical outcomes were measured using the Canine Brief Pain Inventory (CBPI) and Canine Orthopedic Index (COI) before treatment and at 15, 30, 60, 90, and 120 days post-treatment. The Shapiro–Wilk test was used to check for data normality. Group comparisons at each time point were analyzed using the Kruskal–Wallis H test, followed by Dunn's *post-hoc* test, *p* < 0.05. Kaplan–Meier estimators were used to create time-to-event probabilities and curves.

**Results:**

All treatments produced clinically significant improvements. The MG and PRPG produced significant improvements in Pain Severity Scores (PSS) and Pain Interference Scores (PIS) scores up to +90d, but only the PRPG maintained these improvements up to +120d (*p* = 0.03). All groups produced similar results in general gait up to 120 days. No side effects were recorded for any of the treatments.

**Conclusions and Clinical Relevance:**

All three treatments are good options that can be effective, with PRP being the most advantageous, in terms of clinical improvement and duration of effect.

## Introduction

Degenerative lumbosacral stenosis (DLS) is a multifactorial acquired condition, characterized by the narrowing of the vertebral canal and/or intervertebral foramina, which may cause compression of the cauda equina and the nerve roots (1). This compression usually causes back pain and, ultimately, neurological deficits. This disease is most common in large breeds, with the German Shepherd dog (GSD) being the most affected breed, and working dogs, with greater incidence on males (1). Patients suffering from DLS can develop back pain, posterior limb lameness, hyperesthesia, loss of proprioception and urinary/fecal incontinence (2).

In working dogs, lumbosacral disease is a predominate condition leading to early retirement (27% of cases) and, in the worst case, euthanasia (3). Due to the diverse causes and varying severity of DLS, there is no universally accepted treatment protocol. Management options include conservative therapy, surgical intervention, and epidural steroid injections (4). Conservative treatment has approximately 50% success in symptom relief. Surgical treatment has shown improvement in 67%−97% of cases, although the variety of techniques complicates direct comparison.

In many DLS cases inflammation substances persist even after surgical decompression, continuing to cause the symptoms (5). To reduce this inflammation, epidural injections of corticosteroids are commonly used, and methylprednisolone is a common choice (5). With these limitations in therapeutic options concerning effectiveness and possible side-effects, there is a need for more information on better approaches. Local intradermal therapy, also known as mesotherapy, is a technique that involves injecting medication into the superficial layers of the skin. This minimally invasive technique has a rapid onset of action with a prolonged local effect with very limited systemic exposure, as it uses much smaller doses (6). This method allows the drug to act locally in the underlying tissues, even reaching the articulations, with slow absorption and a lower frequency of administrations (7). It has been used to manage painful musculoskeletal conditions in humans, horses, and dogs, particularly for back pain (8–11).

Similarly, autologous platelet therapies, such as platelet-rich plasma (PRP), offer a promising approach due to the crucial role platelets play in the body's natural healing response. PRP is believed to reduce inflammation and support tissue regeneration (12, 13). Platelets products have an anti-inflammatory effect, reducing the effect of pro-inflammatory cytokines, which in turn will reduce pain associated with DLS (4). The use of PRP for treating musculoskeletal conditions in dogs has been extensively documented, with positive outcomes (14). Both PRP and mesotherapy approaches are common procedures in referral rehabilitation centers specializing in pain management and have the advantage of producing long-lasting effects with minimal side effects.

This retrospective study aimed to compare the effectiveness of a mesotherapy protocol with that of PRP or methylprednisolone epidural injection in managing DLS in working police dogs. We hypothesized that both treatments can improve signs in affected dogs, compared to methylprednisolone.

## Methods

The study protocol was approved by the ethical review committee of the Faculty of Veterinary Medicine, Lusófona University (Comissão de Ética e Bem Estar Animal - CEBEA, approval n 22/2025) and complied with the relevant institutional and national guidelines for the care and use of animals. The ARRIVE guidelines for reporting were followed. Written, informed consent was obtained from the Institution responsible for the animals.

### Patient selection

Electronic medical records were searched to identify all dogs with DLS, evaluated at the Canine Veterinary Center of the Guarda Nacional Republicana (Portuguese Gendarmerie). To be included in the study, dogs had to be diagnosed with DLS based on a complete physical and neurological examination (lumbosacral pain or hyperesthesia, pelvic limb paresis or weakness, decreased hind limb withdrawal reflex, and proprioceptive deficits) (15); have radiographic and computed tomography findings consistent with DLS (4); have confirmed absence of any other disease (clearly stated on the medical records, with available copies of additional performed diagnostics); have available results of the Canine Brief Pain Inventory and Canine Orthopedic Index for the set follow-up evaluations (16, 17); have been treated with the same treatment frequency (2 sessions, 2 weeks apart from each other). Additional inclusion criteria included a body weight≥15 kg, age >2 years, and the absence of any other concomitant treatment.

For a treatment to be considered in the study, records of at least 10 animals had to be available. Three treatments were identified: a mesotherapy protocol (MG, *n* = 30), consisting of two sessions spaced 15 days apart (18). The solution administered contained 20 mg of lidocaine, 1 mg of dexamethasone, and 5 mg of midazolam, for a total volume of 3 ml. The second treatment considered was the EI of a single PRP product (CRT PurePRP Kit, Companion Regenerative Therapies, Newark, DE, USA), administered twice, with a 15-day interval between administrations (PRPG, *n* = 15) (4). The third treatment considered was be EI of methylprednisolone (MTPG, *n* = 10), at a dose of 1 mg/kg, administered twice, with a 15-day interval between administrations (4). The flow chart of the process is presented in Figure 1.

**Figure 1:**
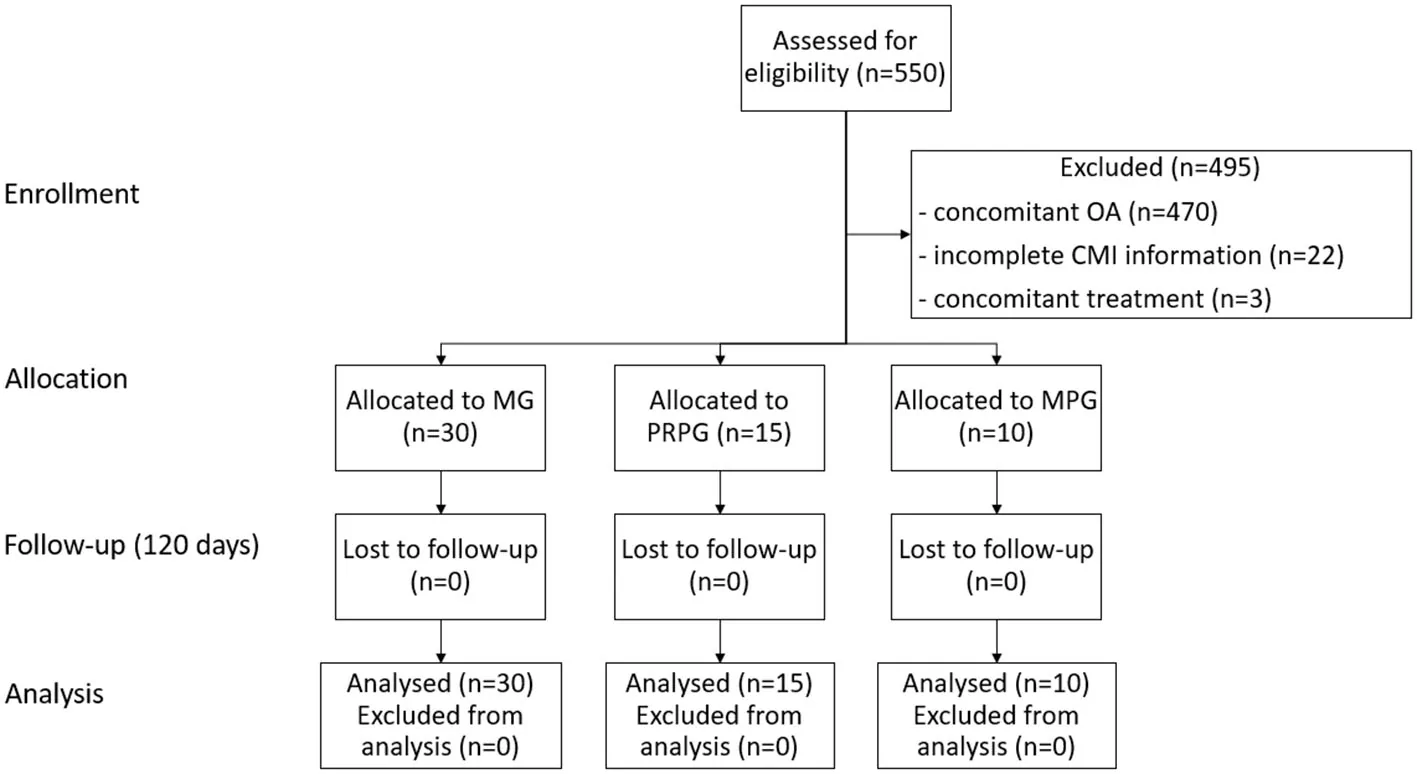
Full study flow diagram. PRPG, platelet-rich plasma group; MG, mesotherapy group; MPG, methylprednisolone group.

### Treatment technique

Treatments were conducted as previously described. In MG, a solution volume of 0.1–0.2 ml was injected intradermally at each injection point. The injection sites were spaced approximately 2 cm apart along the skin area corresponding to the lumbosacral spine. Two injection lines were conducted on each side of the spine (Figure 2). Four millimeter, 27-gauge mesotherapy needles (Mesoram, Italy) were used. Before the injections, the hair and skin were thoroughly scrubbed with a disinfectant solution around the area of interest. The hair was not clipped (19, 20). Two sessions, according to what was previously described (10, 11).

**Figure 2:**
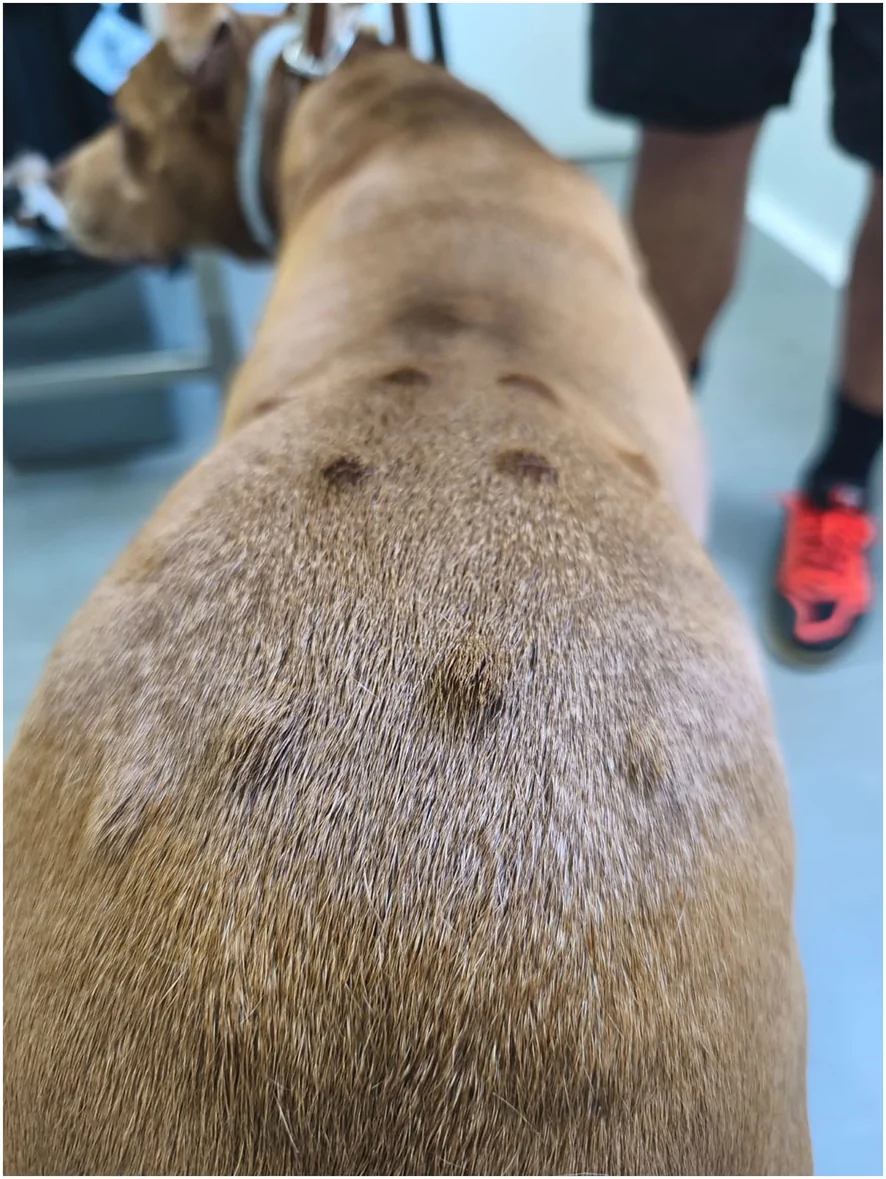
A dog after the mesotherapy session.

Dogs in the PRPG group received two intra-articular (IA) injections of 4 ml of PRP. The PRP was prepared using a commercially available kit (CRT PurePRP^®^, Companion Animal Health by Enovis^®^, Lewisville, TX, USA). The preparation protocol has been described before (12). In brief, 50 ml of whole blood was drawn from the jugular vein into a 60 ml syringe preloaded with 10 ml of Anticoagulant Citrate Dextrose Solution. The collected blood was transferred to a concentrating device and centrifuged at 3,600 rpm for 1 min using the Executive Series Centrifuge II (Companion Animal Health by Enovis^®^, Lewisville, TX, USA). After this first centrifugation, the buffy coat and platelet-poor plasma were harvested and placed into a second concentrating device. A subsequent centrifugation was performed at 3,800 rpm for 5 min. Following the second spin, the volume of platelet-poor plasma was reduced to 4 ml. The device was gently agitated to resuspend the platelets, and the resulting PRP was drawn into a 12 ml syringe. The PRP was used immediately after preparation and was administered without an activating agent. Samples of both PRP and whole blood were sent to an external laboratory for compositional analysis and comparison. Dogs in MPG received a 1 mg/kg dose of methylprednisolone acetate (Depo-medrol, Pfizer, Portugal), injected into the epidural space.

EI was performed under sedation (21), achieved through the simultaneous intravenous administration of medetomidine (0.01 mg/kg) and butorphanol (0.1 mg/kg). Patients were positioned in sternal recumbency with the pelvic limbs extended rostrally. The injection site was, with margins extending laterally to the ilial wings, cranially to L3–4, and caudally to S3-Co1. The skin was prepared aseptically in a standard presurgical fashion. A spinal catheter was then placed in the epidural space near the L7–S1 vertebrae. Accurate needle placement was confirmed using the “hangingdrop” technique with sterile saline (21).

### Outcome measures

Different clinical metrology instruments (CMI) or client-reported outcome measures (CROMs) have been developed to evaluate the severity of orthopedic conditions and the response to treatment in dogs. Still, as pain is a multi-dimensional experience that encompasses more than just a functional aspect, gathering information across several CMIs may help capture this complex experience (22). Response to treatment was be measured using the Canine Brief Pain Inventory and Canine Orthopedic Index before treatment (day 0), and at 15 (+15d), 30 (+30d), 60 (+60d), 90 (+90d), and 120 (+120d). The same dog handler must complete all instruments for each dog throughout the follow-up period. Both instruments have been previously validated in their Portuguese versions (16, 17).

### Statistical analysis

Normality was assessed with the Shapiro-Wilk test. In each evaluation moment, the groups' results were compared using a Kruskal–Wallis H-test, followed by Dunn's *post-hoc* test. The effect size, as the eta squared, was determined and considered to be small if 0.01– < 0.06, medium if 0.06– < 0.14, and large if >0.14. Kaplan–Meier estimators were used to generate time-to-event curves and time-to-event probabilities. Different outcomes were established for each clinical metrology instrument, based on the measures of success that have been defined as constituting a clinically important difference. With the CBPI, it has been set as a reduction of ≥1 in PSS and ≥2 in PIS (23). For the COI, the success level has been determined as a reduction of ≥14 for the overall score (24). For the dimensions of the COI (stiffness, gait, function, and QOL), success has been set as a reduction of −4 for Stiffness, Function, and Gait and −3 for QOL a reduction of ≥3.5 (24). All results were analyzed using commercially available software (IBM SPSS Statistics version 20).

## Results

The sample included 55 dogs, identified out of 550 animals assessed for eligibility, with a mean body weight of 29.9 ± 6.2 kg and a mean age of 8.7 ± 2.8 years. Both sexes were represented, with 32 males and 23 females, and four main breeds were represented: Belgian Malinois Shepherd Dogs (*n* = 25), German Shepherd Dogs (*n* = 13), Labrador Retriever (*n* = 5), and Dutch Shepherd Dogs (*n* = 2). 10 animals from other breeds were also included in the analysis.

The composition of whole blood and PRP is presented in Table 1. Preparation of PRP took around 20 min, from the initial blood collection to the final administration.

**Table 1:** Mean values (±standard deviation) of whole blood and platelet-rich plasma product composition.

Parameter	Whole blood		Platelet concentrate	
	Mean Value	SD	Mean Value	SD
Platelets (x10^3^/mm^3^)	341.05	90.44	1828.12	476.26
RBC (x10^6^/mm^3^)	7.57	1.36	0.49	0.06
WDC (x10^3^/mm^3^)	11.40	4.49	4.96	3.81
Linfocytes (x10^3^/mm^3^)	2.46	0.92	2.84	1.85
Monocytes (x10^3^/mm^3^)	0.80	0.45	0.62	0.38
Neutrophils (x10^3^/mm^3^)	8.74	3.70	0.86	0.35
Eosinophils (x10^3^/mm^3^)	0.42	0.52	0.39	0.45
Basophils (x10^3^/mm^3^)	0.01	0.02	0.00	0.00

No clinically-significant differences were observed at T0 regarding CROMs (CMIs) scores, age, bodyweight, and distribution of breed, age or sex between groups. CROM scores in each group are presented in Table 2. Regarding the CBPI, differences were observed in PSS and PIS, from +15d up to +120d. Results with PSS showed clinically significant improvements with MG and PRPG until +90d. At the final follow-up, only PRPG had such improvements. At +15d, differences between the groups were due to a greater effect of PRPG (*p* = 0.03 compared to MG, and *p* = 0.02 compared to MPG), and differences between PRPG and MG (*p* < 0.01) at +30d, at +60d (*p* < 0.01), and +120d (*p* = 0.03). The changes in PSS scores are represented in Figure 3. A similar trend was observed with PIS, with clinically significant improvements with MG and PRPG until +90d, and only PRPG at +120d. At +15d, differences were due to a greater effect of PRPG (*p* < 0.01 compared to MG, and *p* = 0.01 compared to MPG), and differences between PRPG and MG (*p* < 0.01) at +30d, at +60d (*p* < 0.01), and +120d (*p* = 0.01).

**Table 2:** Variation of the considered client-reported outcome measures (median, inter-quartile range, and percentual change) by group and moment.

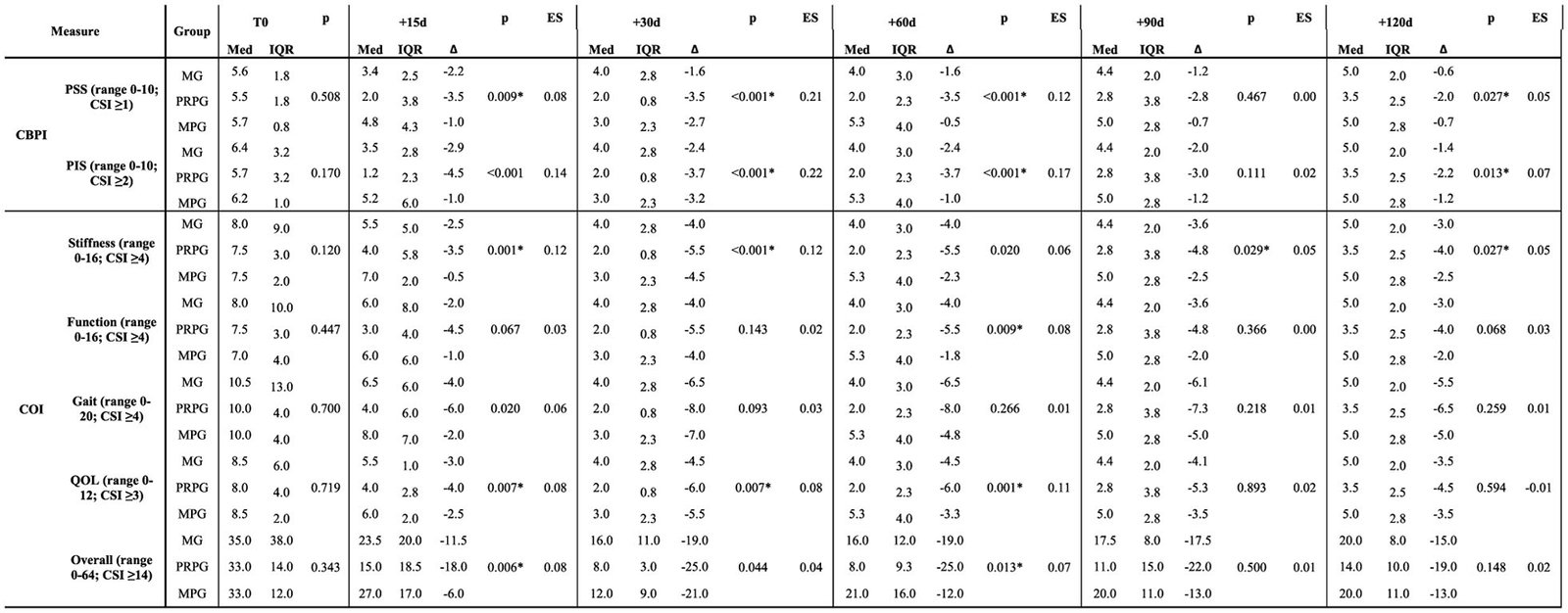

CBPI, Canine Brief Pain Inventory; COI, Canine Orthopedic Index; CSI, Clinical significant improvement; PIS, Pain Interference Score; PSS, Pain Severity Score; QOL, Quality of Life. ^*^indicates significance when comparing groups at each follow-up moment. The effect size is small if 0.01− < 0.06, medium if 0.06− < 0.14, and large if >0.14.

**Figure 3:**
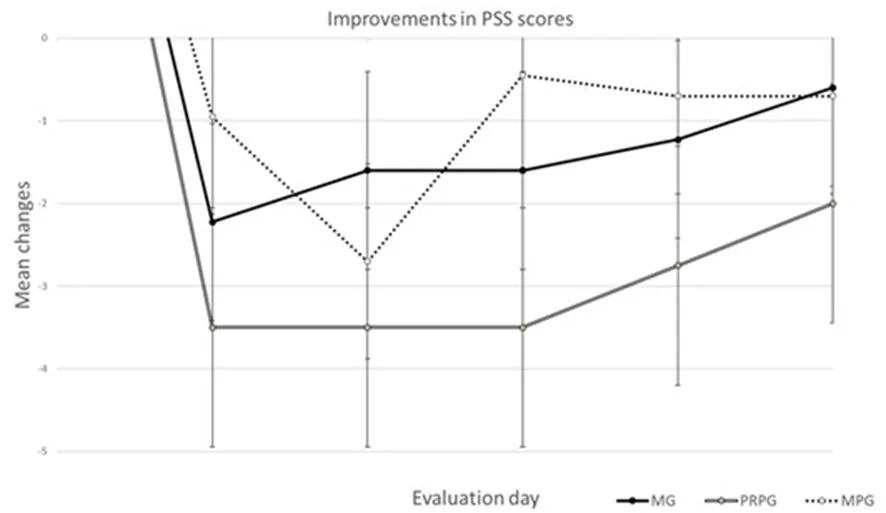
Mean change in Pain Severity Score (PSS) per group from baseline (±SEM) in the considered evaluation days. With the PSS, a reduction of 1 or more is considered clinically significant.

MG and PRPG produced clinically significant improvements of stiffness scores up to +60d and +120d, respectively. PRPG was better able to decrease stiffness scores, with differences observed at +15d, (*p* = 0.02 compared to MG, and *p* < 0.01 compared to MPG), +30d, (*p* < 0.01 compared to MG, and *p* = 0.04 compared to MPG), and between PRPG and MG at +90d (*p* = 0.03). With Function, MG and PRPG also produced clinically significant improvements up to +60d and +120d, respectively. Significant differences were only observed between PRPG and MG (*p* = 0.01) at +30d. Regarding Gait, all groups were able to produce improvements up to the last evaluation point (+120d), at a similar level. For QOL, MG and PRP produced great differences for a longer period. At +15d PRPG performed better than MPG (*p* = 0.01), and also at +60d (*p* = 0.01). With the overall COI score, a positive trend was observed, with all groups producing clinically significant improvements until +120d. At +15d PRPG performed better than MPG (*p* = 0.01), and at +90d, PRPG than MG (*p* = 0.03). The changes in overall COI scores are represented in . Most differences were medium to large.

Results of the Kaplan–Meier estimators with each instrument are shown in Table 3. Better results for more extended periods with the considered instruments were observed in PRPG, where a longer period was required for scores to drop below the clinically significant improvement level. No side effects were recorded.

**Table 3:** Time (in days) to record a first reduction below clinically significant levels, calculated with Kaplan–Meier estimators and compared with the Log–rank test.

Outcome Measure		Log Rank test	Group											
				MG				PRPG				MPG			
			Mean	SD	95% CI		Mean	SD	95% CI		Mean	SD	95% CI	
CBPI	PSS	0.009^*^	91.3	9.2	53.3	89.3	102.5	4.3	24.1	40.9	50.0	15.1	20.5	79.5
	PIS	0.003^*^	90.0	8.0	54.2	85.8	105.5	4.4	26.8	44.2	32.5	11.3	10.4	54.6
COI	Stiffness	0.011^*^	57.0	8.3	30.7	63.3	35.5	4.5	26.7	44.3	57.5	1.7	14.2	20.8
	Function	0.002^*^	92.0	8.8	34.7	69.3	95.5	4.5	26.7	44.3	87.5	1.7	14.2	20.8
	Gait	< 0.001^*^	100.0	9.2	61.9	98.1	118.5	2.4	13.7	23.3	105.0	14.3	22.0	78.0
	QOL	0.021^*^	96.6	8.3	9.7	80.8	91.8	0.8	13.6	7.8	36.6	7.6	11.9	12.3
	Overall	< 0.001^*^	111.0	7.5	26.3	55.7	118.5	2.4	13.7	23.3	55.0	5.0	15.0	15.0

^*^indicates significance.

## Discussion

In this study, both the mesotherapy protocol and the administration of platelet-rich plasma effectively improved clinical signs, supporting the initial hypothesis. The use of PRP resulted in more pronounced and prolonged improvements across pain and functional scores compared to both mesotherapy and epidural methylprednisolone.

When evaluating the changes in pain parameters, both MG and PRPG produced clinically significant improvements in PSS and PIS up to +90d post-treatment, PRPG maintained these up to the final follow-up at +120d. So, dogs in the PRPG not only saw the most improvement, but this also lasted the longest amongst all groups. The more marked and longer lasting efficacy of PRP is likely attributed to the capability of platelets to regenerate damaged tissues and not only alleviate the pain and inflammation (25). Being composed of the patients own cells, it also decreases the risk of adverse reactions and the concerns regarding possible long term use.

Regarding functional and mobility changes reflected in the COI, distinct variations were observed between the treatments. Both MG and PRPG produced improvements in stiffness and overall function up to +60d and +120d, respectively. PRPG improved stiffness scores more, compared to MG and MPG during the early evaluation phases (+15d and +30d). All three groups were able to produce improvements in gait up to +120d at a similar level, but the targeted regenerative nature of PRP provides a much more profound impact on specific indicators, like QOL and stiffness.

Some considerations need to be made to the treatment procedure itself, as it can influence the decision making. One is the ease of each treatment, since MPG and PRPG require sedation, while MG does not. Another, is treatment frequency, particularly regarding MG. For the management of back pain, 2 sessions, 15 days apart, have been recommended in dogs and horses (10, 11). In contrast, more sessions have been described for the management of other chronic painful conditions, as osteoarthritis (19, 20, 26). Is the possible that this last protocol can be more effective, and it should be evaluated in future studies.

This study also has some limitations, partly related to its retrospective nature. There were differences in group sizes (*n* = 30 for MG, *n* = 15 for PRPG, and *n* = 10 for MPG), which can affect the representation when comparing the treatments. The study population consisted exclusively of working police dogs and predominantly Belgian Malinois and German Shepherd Dogs. The high physical demands and specific anatomical particularities of this group mean the findings may vary for the standard pet dog and even achieve better results, due to less intensive exercise (27, 28). Future prospective studies should include sample power calculations, the inclusion of a negative control group, and objective measures, such as a pressure-sensitive walkway, stance analysis, or thermographic evaluations (29), to complement CMI information. A longer follow-up period would also be of interest to determine the longevity of each treatment and the need for follow-up treatments.

## Conclusions and clinical relevance

Mesotherapy, epidural injection of platelet-rich plasma or methylprednisolone proved to be safe, viable options with no side effects for managing degenerative lumbosacral stenosis in highly active working dogs. However, the treatment with PRP seems to be the one achieving more clinical improvements for longer, offering a better and durable outcome, not only of pain relief, but also functional scores.

## Data Availability

The raw data supporting the conclusions of this article will be made available by the authors, without undue reservation.
